# Pilomatrixoma of the Forearm in an Elderly Male

**DOI:** 10.7759/cureus.54623

**Published:** 2024-02-21

**Authors:** Víctor García Rodríguez, Maribel Iglesias Sancho, Noelia Pérez Muñoz, Montse Salleras Redonnet

**Affiliations:** 1 Dermatology, Hospital Universitari Sagrat Cor, Grupo Quirónsalud, Barcelona, ESP; 2 Pathology, Hospital Universitari General de Catalunya, Grupo Quirónsalud, Sant Cugat del Vallés, ESP

**Keywords:** histopathology examination, ultrasound imaging, dermoscopy image analysis, diferential diagnosis, adnexal skin tumor

## Abstract

Pilomatrixoma or pilomatricoma is a benign adnexal neoplasm originating from the hair matrix, the inner sheath of the hair follicle, and the hair cortex. Although it is considered rare in adults, numerous cases have been documented in the literature. We present a case of an elderly male who sought consultation due to a newly appearing nodular lesion on his left forearm. Several benign and malignant entities were included in the original differential diagnosis. High-frequency ultrasonographic features suggested a cystic neoplasm with calcification and mild intralesional vascularity. Ultimately, histopathological examination confirmed the diagnosis of pilomatricoma. In this study, our aim is to review the importance of the available diagnostic tools, such as dermoscopy, and the emerging utility of cutaneous high-frequency ultrasonography. Some rarer pathological variants are also discussed, including perforating, anetodermic, bullous and pigmented pilomatricoma. We hope that exposure to these clinical, dermoscopic, ultrasonographic, and histopathological images will encourage clinicians to consider pilomatricoma in their differential diagnosis when approaching nodular lesions, regardless of location and patient’s age.

## Introduction

Pilomatrixoma (PM) or calcifying epithelioma of Malherbe is a benign adnexal neoplasm, which derives from the hair matrix, the inner sheath of the hair follicle, and the hair cortex. Although PM is the most common adnexal neoplasm in children, its age range spans from three months to 93 years old. It is slightly more frequent in females. Lesions may be solitary (more frequent) or multiple, in which case some syndromes must be ruled out, such as Steinert myotonic dystrophy (myotonia, skeletal muscle atrophy, dysarthria, drooping eyelids, etc.), adenomatous polyposis-related syndromes, like Gardner syndrome; gliomatosis cerebri, Turner syndrome, Rubinstein-Taybi syndrome and trisomy 9, among others [1-3]. PM mainly favors the head and neck area but can appear on the trunk and extremities and usually consists of pinkish-bluish firm nodules [4,5]. Diagnosis is made by clinical evaluation and histopathological confirmation. However, dermoscopy and high-frequency ultrasound (HFUS) have proven to be useful in the diagnostic process.

## Case presentation

**Figure 1:**
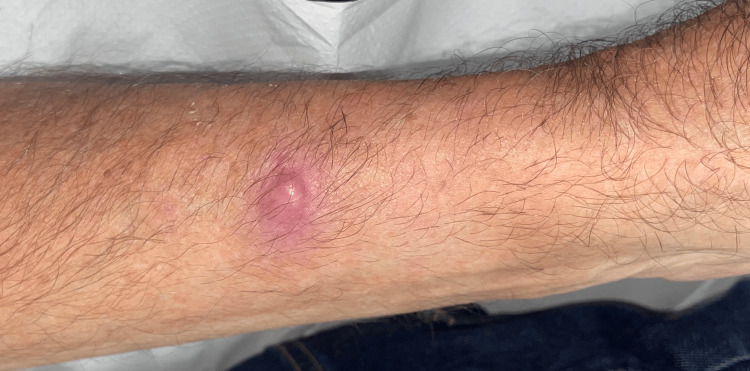
Clinical picture of the nodule The firm pink nodule measuring 1x1 cm on the radial aspect of the left forearm.

A man in his 70s consulted at our Department because of a new-onset asymptomatic nodule on his left forearm. Physical examination demonstrated an erythematous-yellowish mobile nodule, mobile and of firm consistency (Figure 1).

**Figure 2:**
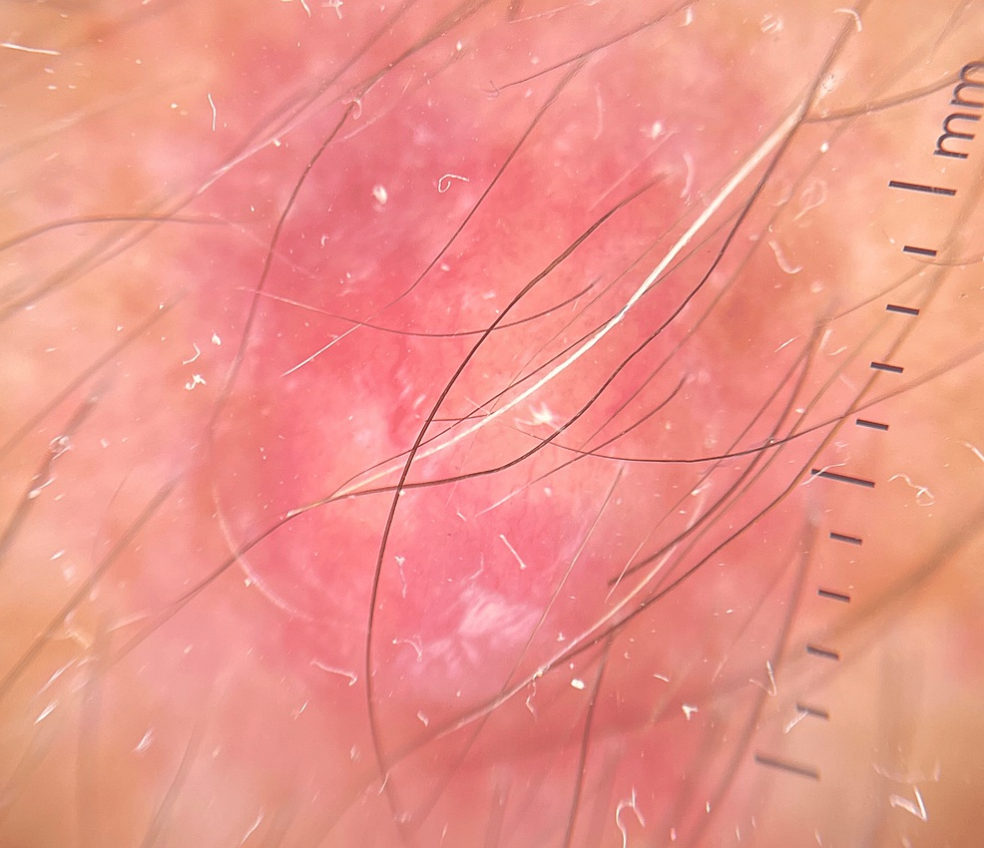
Polarized dermoscopy image Erythematous background with yellowish areas and visible dilated irregular vessels. White streaks can also be noted. (Dermoscopy was performed using the DermLite DL200 Hybrid dermatoscope (DermLite LLC, Orange County, CA), 10X magnification).

Dermoscopy showed an erythematous background with yellowish and white areas, accompanied by white streaks and irregular linear vessels (Figure 2).

The differential diagnosis included pseudolymphoma, nodular hidradenoma, cutaneous metastasis, lymphoma, and benign or malignant cystic neoplasm, such as infundibulocystic basal cell carcinoma.

**Figure 3:**
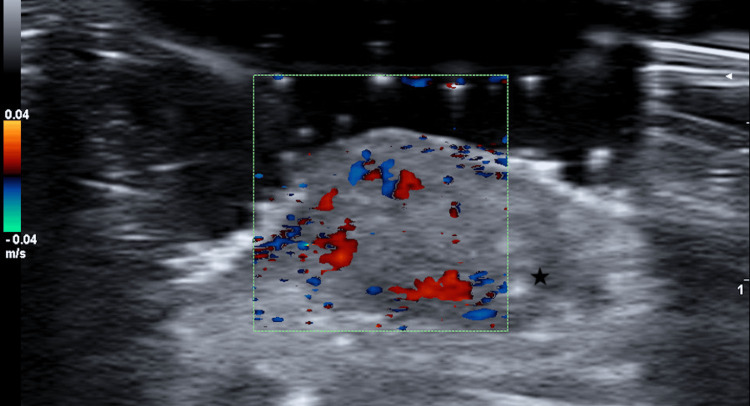
High-frequency ultrasonographic assessment image Round to oval hypoechoic dermal lesion with hyperechoic dots (star) and intralesional vascularization on color Doppler assessment. High-frequency ultrasonographic assessment was done with ESAOTE MyLab Gamma™, 18MHz probe (Universal Diagnostic Solutions, Inc., La Mirada Dr. Vista, CA).

HFUS assessment with an 18MHz probe (ESAOTE MyLab Gamma™; Universal Diagnostic Solutions, Inc., La Mirada Dr. Vista, CA) showed a well-defined round to oval hypoechoic dermo-hypodermic lesion with hyperechogenic dotted foci and moderate intralesional color Doppler signal (Figure 3).

**Figure 4:**
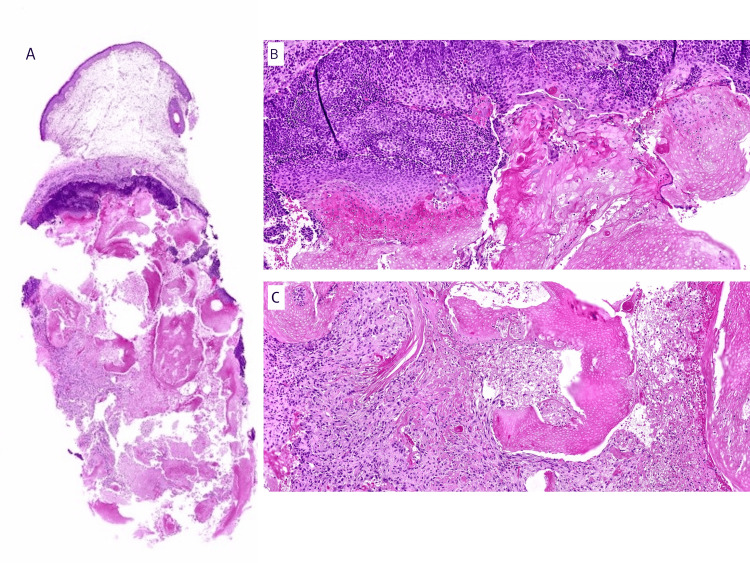
Histopathological examination of a 4-mm cutaneous punch biopsy A. 60x; Relatively well-demarcated dermal lesion. B. 100x; Three different cellular components can be seen: basaloid matrical and supramatrical cells transitioning towards more eosinophilic nucleate cells with squamoid appearance. Lastly, cornified cells show only nuclear outlines and anucleate cells known as ghost cells. C. 100x; Keratin debris admixed with histiocytic inflammation. Hematoxylin and Eosin (H&E) stain was used for histopathology study.

A 4-mm punch biopsy was performed for histopathological evaluation. Hematoxylin and eosin sections showed a dermal lesion composed of basaloid nests resembling those of the hair matrix, squamoid cells, and ghost cells, together with a histiocytic reaction (Figures 4A-4C).

These findings were diagnostic of PM. The histopathological study did not show calcification despite hyperechogenic foci on HFUS, although it is worth noting it was an incisional punch biopsy. The nodule was successfully excised without posterior recurrence.

## Discussion

PM is the most frequent adnexal neoplasm in children [5]. Despite a bimodal age distribution (0-20 years, 50-65 years), the vast majority appear under 10 years of age and are mostly located on the face, neck, or upper trunk [6]. The ears, eyelids, breasts, and fingers have also been described as unusual locations [4,5]. PM located on the forearm has also been reported [7]. In its pathogenesis, somatic mutations in exon 3 of the *CTNNB1* gene, which encodes b-catenin (a protein involved in Wnt signaling), might be involved [8]. *BCL2* could also play a pathogenetic role [9]. PM has also been reported to appear at coronavirus disease 2019 (COVID-19) vaccination sites [10,11]. Germline mutations in *PLCD1* have been reported in multiple PM [12]. Multiple familial PM combined with polyposis should raise suspicion to rule out Gardner syndrome. Associated midfacial vascular stains, keloids, broad thumbs/halluces, and hypertrichosis prompt screening of Rubinstein-Taybi syndrome [1].

Dermoscopic features include an erythematous background, diverse vascular patterns (dotted, irregular, and hairpin vessels), homogeneous white areas, white streaks, bluish areas, and ulceration [13,14]. Some of the aforementioned characteristics were present in our patient. However, these findings are rather nonspecific and do not 100% rule out malignancy. HFUS most commonly shows round to oval heteroechoic nodules with internal echogenic foci and a hypoechoic rim [15,16] and is potentially of great help in unusual locations, as in our case.

From the histopathological perspective, several variants have been reported. Transepidermal elimination of connective tissue may be noted and should be interpreted as a secondary perforating disease [17]. PM may develop secondary overlying anetoderma [16]. A bullous version of PM, although controversial, has also been described [11]. Pigmented PM, characterized by the presence of melanocytic hyperplasia and/or melanin pigments within the basaloid cells, may appear in over 25% of the cases, according to a clinicopathological review of 57 cases [18], although it is worth noting that all of the patients were Japanese. Some PM have amyloid deposits without further clinical implications, which were not detected in our patient. Calcification is common and is said to be more prominent in fully developed PM. However, this hypothesis has not been completely demonstrated since calcification can occur at any stage of the lesion.

Differential diagnosis with other benign and malignant entities is sometimes difficult owing to the heterogeneity in clinical manifestations, with some series showing pre-procedural diagnostic suspicion in under a third of the cases [4]. A broad differential diagnosis was discussed in our case, but HFUS helped us narrow the plausible options, and histopathology confirmed the diagnosis of PM.

Standard care involves simple excision, with good results and low recurrences. There is a potential risk of malignancy in the form of pilomatricial carcinoma. A strong association, however, has not been robustly demonstrated. Although PM might show mitosis to a reasonable degree, these are much more abundant in pilomatricial carcinoma, accompanied by nuclear pleomorphism.

## Conclusions

Herein, we present a case of a PM located on the forearm of an elderly patient, and underscore the utility of clinical, dermoscopic, ultrasonographic, and histopathological correlation. Histopathology remains the gold standard for diagnosis. Awareness of unusual clinical and histopathological variants of PM may aid in the differential diagnosis and reduce morbidity. Surgical excision remains the mainstay treatment, usually with low recurrence rates.
